# *MAD1L1* Arg558His and *MAD2L1* Leu84Met interaction with smoking increase the risk of colorectal cancer

**DOI:** 10.1038/srep12202

**Published:** 2015-07-17

**Authors:** Rong Zhong, Xiaohua Chen, Xueqin Chen, Beibei Zhu, Jiao Lou, Jiaoyuan Li, Na Shen, Yang Yang, Yajie Gong, Ying Zhu, Jing Yuan, Xiaoping Xia, Xiaoping Miao

**Affiliations:** 1 Department of Epidemiology and Biostatistics, and the Ministry of Education Key Lab of Environment and Health, School of Public Health, Tongji Medical College, Huazhong University of Science and Technology, Wuhan 430030, Hubei, China; 2 Department of Laboratory Medicine, No 161 Hospital of PLA, Wuhan 430010, China; 3 Department of Medical Genetics, school of Basic Medical Science, Wuhan University, Wuhan 430071, China; 4 Department of Occupational and Environmental Health, School of Public Health, Tongji Medical College of Huazhong University of Science and Technology, Wuhan 430030, Hubei, China; 5 Clinical Laboratory of the Fourth Affiliated Hospital, Zhejiang University School of Medicine, Yiwu, China

## Abstract

The spindle assembly checkpoint (SAC) has been established as an important mechanism of driving aneuploidy, which occurs at a high frequency in the colorectal tumorigenesis. Two important components of SAC are MAD1L1 and MAD2L1, which function together in an interactive manner to initiate the checkpoint signal. We hypothesize that genetic variants in the binding domains of MAD1L1 and MAD2L1 may modulate protein structures and eventually contribute to CRC susceptibility. A case-control study including 710 CRC cases and 735 controls was performed to examine *MAD1L1* Arg558His and *MAD2L1* Leu84Met’s conferring susceptibility to CRC. Cytokinesis-block micronucleus cytome assays were applied to assess the effect of two functional variants on chromosomal instability (CIN). Significant associations with CRC risk were observed for *MAD1L1* Arg558His (OR = 1.38,95% CI: 1.09–1.75) and *MAD2L1* Leu84Met in a dominant model (OR = 1.48,95% CI: 1.09–2.01). Moreover, significant multiplicative gene-smoking interactions were found in *MAD1L1* Arg558His (*P* = 0.019) and *MAD2L1*84 Leu/Met (*P* = 0.016) to enhance CRC risk. Additionally, the frequencies of lymphocytic micro-nucleated binucleated cells for *MAD1L1* Arg558His polymorphism were significantly different in the exposed group (*P* = 0.013), but not in the control group. The study emphasized that *MAD1L1* Arg558His and *MAD2L1* Leu84Met can significantly interact with smoking to enhance CRC risk, and the genetic effects of MAD1L1Arg558His on CIN need to be further clarified in follow-up studies.

Colorectal cancer (CRC) remains the third most commonly diagnosed cancer and the fourth leading cause of cancer mortality worldwide, accounting for 8.3% of the total cancer cases and 6.3% of cancer deaths^1^. With the progressive “Westernization” of lifestyles, the incidence of CRC seems to have rapidly become an epidemic in Asian, especially in China. CRC has been established as a complex disease that is strongly influenced by multiple genetic and environmental factors and their complex interactions. Genetic factors play a decisive role in the development of CRC when only a fraction of exposed individuals actually develop CRC during their lifetimes.

Genome instability is the prominent hallmark of human cancers and of chromosomal aneuploidy, which is the most common type of genomic instability, occurring at a high frequency in the colorectal tumorigenesis^2^,^3^. The spindle assembly checkpoint (SAC) is especially essential to ensure accurate chromosome segregation and to prevent the formation of aneuploidy^4^,^5^. However, a malfunction of the SAC is an important mechanism of driving chromosomal aneuploidy, which advances the carcinogenesis. Evidence from molecular analyses has shown that the proper function of SAC is highly dependent on the strictly orchestrated expression of its components, and an increase or decrease in one of the functional components may lead to the process of an aberrant spindle checkpoint.

Two interactive components of the SAC are the human MAD1L1 mitotic arrest deficient-like 1(MAD1L1) and MAD2L1 mitotic arrest deficient-like 1(MAD2L1), which function together in a hetero-tetrameric complex to initiate the checkpoint signal^6^,^7^. First, MAD1L1 has a crucial role in the activation and localization of MAD2L1 to unattached kinetochores. Additionally, MAD2L1 is an important component of the anaphase-promoting complex or cyclosome (APC/C) inhibitory complex^8^. In fact, MAD2L1 has been shown to inhibit the activity of APC/C through directly binding to CDC20, and eventually to regulate the metaphase-anaphase transition until sister chromatids achieve proper alignment and microtubule attachment^9^,^10^. A depletion of MAD1L1 in mammalian cells has been found to inhibit the localization of MAD2L1 to kinetochores, and a disruption of MAD2L1 may not effectively bind to CDC20 to regulate the metaphase-anaphase transition^11^,^12^. Taken together, the structure changes or the loss of MAD1L1 and MAD2L1 may severely disturb the spindle checkpoint function, leading to aneuploidy and carcinogenesis^13^,^14^.

Therefore, the binding domains of MAD1L1 and MAD2L1 are required for transducing the checkpoint signal, and any change in the gene structure may disturb the process of checkpoint signal transduction. Genetic variants in the binding domains of MAD1L1 and MAD2L1 may modulate protein structures and eventually contribute to CRC susceptibility. *MAD1L1* Arg558His (rs1801368) is a missense variant at codon 558 that is located in the region that encodes the second leucine zipper domain of MAD1L1, and the His allele of *MAD1L1* Arg558His was identified to reduce the binding activity of MAD2L1 to MAD1L1, with the result of a decreased proficiency in enforcing mitotic arrest. Moreover, *MAD1L1* Arg558His has been previously reported to be associated with lung cancer risk^15^,^16^. In addition, the other missense polymorphism resulting in Leu^84^ to Met^84^ substitution in the *MAD2L1* molecule has also been found to influence the interaction between MAD1L1 and MAD2L1^16^. Considering the functional missense variants *MAD1L1* Arg558His and *MAD2L1*, Leu84Met may play an important role in transducing the checkpoint signal by potentially influencing the protein activity. An alternative hypothesis has been proposed that *MAD1L1* Arg558His and *MAD2L1* Leu84Met may be associated with the risk of CRC.

Biological evidence has indicated that the *MAD1L1* Arg558His and *MAD2L1* Leu84Met variants may result in a mitotic check-point defect by severely disturbing the domains of MAD1L1 and MAD2L1, directly leading to chromosomal instability (CIN). It has been clear that CIN is the important phenotype of the carcinogenesis and that it plays a causative role in tumor initiation and progression in CRC biology. Consequently, not only were epidemiology studies conducted to explore the association between the two variants and the risk of CRC, but also cytokinesis-block micronucleus cytome assays (CBMN) have been applied to examine the effect of two functional genetic variants on chromosomal instability (CIN). Here a case-control study consisting of 710 CRC cases and 735 controls was performed to examine *MAD1L1* Arg558His or *MAD2L1* Leu84Met conferring susceptibility to CRC in a Chinese Han population. Moreover, the frequencies of lymphocytic micronucleated binucleated cells (MNBNCs) in peripheral lymphocytes was used to assess the effect of the two candidate SNPs on the formation of aneuploidy.

## Results

### Subject characteristics

The baseline characteristics of 710 CRC patients and 735 controls are shown in Table 1. The cases and controls were matched well on the distribution of gender (*P* = 0.520) and age group (*P* = 0.228). Smoking was significantly associated with an increased risk of CRC (*P* < 0.001), with 64.1% of them being smokers and 54.6%, controls. Furthermore, a significant difference in the smoking level was observed between the case and the control groups (*P* < 0.001). Among the smokers, the heavy smokers—who smoked >24 packs per year—were overrepresented compared with the controls (64.0% vs 49.1%). Compared with nonsmokers, the OR for smokers was 1.95 (95% CI = 1.49–2.54).

### Association analysis of candidate SNPs with CRC risk

The genotype information of the two candidate SNPs are presented in Table 2. The genotype distributions of the two SNPs in the controls conformed to the Hardy-Weinberg equilibrium (*P* = 0.872 for *MAD1L1* Arg558His, *P* = 0.311 for *MAD2L1* 84Leu/Met). Minor allele frequencies (MAF) of *MAD1L1* Arg558His and *MAD2L1* 84Leu/Met were 0.461 and 0.059 in controls and 0.524 and 0.087 in cases, respectively. In the logistic regression analysis, the two candidate SNPs were independently associated with CRC risk after adjusting for age, sex, and smoking status. Compared with individuals carrying the *MAD1L1*Arg/Arg genotype, individuals with Arg/His genotype presented a significantly increased risk of CRC on the borderline level (OR = 1.24, 95% CI: 0.96–1.60), but a significant association with CRC risk was observed for individuals with the His/His genotype, with OR of 1.70 (95% CI = 1.26–2.28). For *MAD2L1* 84Leu/Met SNP, heterozygous or homozygous variant genotypes significantly increased the CRC risk compared with the homozygous wild type in dominant genetic models, with OR equal to 1.48 (95% CI: 1.09–2.01).

Table 3 shows the combined effect of the *MAD1L1* Arg558His and *MAD2L1* 84Leu/Met on the risk of CRC. Compared to individuals who carried the combination of *MAD1L1* Arg/Arg and *MAD2L1* Leu/Leu genotypes, we observed that individuals with *MAD1L1* His/His and *MAD2L1* Leu/Met or Met/Met genotypes had an increased risk of OR for CRC risk, up to 2.92 (95% CI: 1.63–5.24). However, no significant multiplicative or additive gene-gene interactions were observed between *MAD1L1* Arg558His and *MAD2L1* 84Leu/Met on the risk of CRC.

### Stratified and interaction analysis for candidate SNPs with CRC risk

In the present study, the stratified analysis and multiplicative and additive interaction analyses were performed to explore the relationship between the two SNPs and smoking (Table 4). When stratified by smoking status, smokers with MAD1L1 Arg/His or His/His genotypes exhibited an increased risk of CRC compared with nonsmokers carrying MAD1L1 Arg/Arg genotype, with an OR of 2.88 (95% CI: 1.91–4.35). Interestingly, the association was even more prominent in heavy smokers when stratified by smoking level, with OR equal to 3.96 (95% CI: 2.56–6.13) for heavy smokers with MAD1L1 Arg/His or His/His genotypes compared with nonsmokers with MAD1L1 Arg/Arg genotype. For MAD2L1 84Leu/Met genotype, smokers carrying Met-containing genotypes were observed to confer a 2.63-fold increased risk of CRC compared with nonsmokers with wide genotypes (95% CI: 1.71–4.05). Similarly, the risk of CRC was increased to 3.48 (95% CI: 1.96–6.21) in heavy smokers when stratified by smoking level. In addition, significant multiplicative but not additive gene-smoking interactions were observed for MAD1L1 Arg558His and MAD2L1 84Leu/Met to enhance the risk of CRC, with P for multiplicative interaction equal to 0.019 and 0.016, respectively.

### Genetic effects of *MAD1L1* and *MAD2L1* on MNBNCs frequency

**Figure 1:**
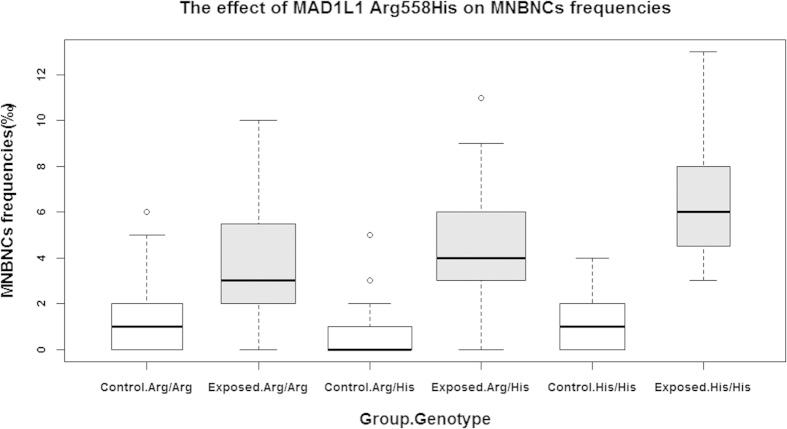
Boxplot of the effect of MAD1L1 Arg558His on MNBNCs frequencies. The *P* values of MNBNC frequencies for individuals with *MAD1L1* His/His, Arg/His and Arg/His genotypes in the control group and the exposed group were 0.580 and 0.013, respectively.

Both MNBNC frequency and genotypes for the two candidate SNPs were examined in the exposed group and the healthy group. Figure 1 shows the effect of MAD1L1 Arg558His on MNBNC frequencies in the exposed and the control groups. No significant statistical difference of MNBNC frequencies was observed for MAD1L1 Arg558His in the control group (*P* = 0.580); however, a significant result emerged in the exposed group. MNBNC frequencies for individuals with *MAD1L1* His/His, Arg/His genotypes and Arg/His genotypes in the exposed group were 3.83 ± 2.58(‰), 4.32 ± 2.68(‰) and 6.67 ± 2.99(‰), respectively, with the *P* value equal to 0.013. Furthermore, negative results were also found for *MAD2L1* Leu84Met in both the exposed and the control groups, with no significantly different distribution of MNBNC frequencies for the three genotypes.

## Discussion

In this study, we conducted a case-control study to explore the association of *MAD1L1* Arg558His or *MAD2L1* Leu84Met with the risk of CRC in a Chinese population. The study demonstrated that both *MAD1L1* Arg558His and *MAD2L1* Leu84Met significantly increased the risk of CRC. Moreover, significant multiplicative gene-smoking interactions were found for *MAD1L1* Arg558His and *MAD2L1* 84Leu/Met to enhance the risk of CRC. In addition, a significant statistical difference was observed for the effect of *MAD1L1L1* Arg558His on the formation of aneuploidy in the exposed group, but not in the healthy control group.

The spindle checkpoint is an intricate multi-protein network that regulates the attachment of sister chromatids to the mitotic spindle in the metaphase to anaphase transition. The cell will proceed to aneuploidy when improper attachments of chromosomes to the spindle microtubules happen. Previous evidence has shown that the chromosomal instability that is induced by the deregulation of the mitotic checkpoint gene can been widely observed in colorectal cancers^17^,^18^,^19^. MAD1L1L1and MAD2L1L1 are two important proteins involved in the spindle assembly checkpoint. Biological function analysis has shown that *MAD1L1* Arg558His cause a deficient metaphase arrest in normal cells^15^. Moreover, a recent study by Guo has also indicated that *MAD2L1* 84Leu/Met may reduce the spindle checkpoint function by physically affecting the interaction of MAD2L1 with MAD1L1 as illustrated via the Co-IP assays^16^. Therefore, the two missense variants in the binding domains of MAD1L1 and MAD2L1 exert the function of disturbing the binding activity of MAD1L to MAD2L1. Consequently, our present epidemiological investigation is consistent with the biological evidence, which indicates that *MAD1L1* 558His and *MAD2L1L1* 84Met are associated with the increased risk of CRC. However, an association of SAC components on CRC risk have not appeared from much larger GWAS studies. It can be given the explanations that the GWAS approach relies on randomly selected SNPs across the genome as markers, most of the associated SNPs identified thus far are unlikely to be the actual causal variants. Besides, in order to prevent false positives associated, GWAS analysis using very stringent P values, it is possible to miss some of the *P* value which is not reached GWAS request is indeed associated SNP^20^,^21^. Furthermore, this case-control study first examined the interaction of *MAD1L1* Arg558His and *MAD2L1* Leu84Met with smoking to increase the risk of CRC. One previous finding from De Voer *et al*. indicated that germ-line mutations in the SAC genes *BUB1* and *BUB3* are associated with an increased risk of CRC^19^. However, in another previous study conducted by Aclavicek *et al*.^22^, the genetic variations in the major mitotic checkpoint genes, including *MAD1L1* and *MAD2L1*, were found not to be significantly associated with the risk of familial breast cancer risk. Further epidemiology studies can also be performed to confirm the results and to estimate the effect of the two missense variants on the risk of other cancers.

MAD2L1 and MAD1L1 are two interactive proteins that are involved in stimulating and executing spindle checkpoint processes. Although biological analyses have shown an attenuated interaction between *MAD1L1* Arg558His and *MAD2L1* Leu84Met, in the present study, we observed no significant interaction between them with regard to the risk of CRC, and a similar result was also found on the risk of lung cancer in a previous study^16^. Similar explanations were also addressed about the discrepancy^16^. Nevertheless, further independent comprehensive studies with larger sample sizes are warranted to clarify the results.

In the present study, the most significant finding was that smoking may interact with *MAD1L1* Arg558His and *MAD2L1* 84Leu/Met to enhance the risk of CRC. Smoking has been firmly established as an important causal factor for the development of CRC by a mass of evidence^23^,^24^. So far, our search in published literature did not yield any evidence of a direct biological function involved in the interaction between *MAD1L1* Arg558His, *MAD2L1* 84Leu/Met and smoking. However, it has been reported that the important specified carcinogens of cigarette smoke, nicotine and polycyclic aromatic hydrocarbons may induce the process of genomic instability by various molecular mechanisms^25^,^26^,^27^,^28^. The malfunction of the SAC plays a prominent role in genomic stability, especially in the development of CRC. The missense variants of the important mitotic checkpoint gene, which may disturb the spindle checkpoint function, seem plausible to interact with smoking to increase the risk of CRC considering the active effect of the cigarette smoke on the process of genomic instability. However, biological functional analyses remain warranted to dissect the molecular mechanism underlying the significant interaction between *MAD1L1* Arg558His, *MAD2L1* 84Leu/Met and smoking.

Lastly, the study explored the effect of *MAD1L1* Arg558His or *MAD2L1* 84Leu/Met on the formation of aneuploidy. Remarkably, MNBNC frequencies for individuals with *MAD1L1* His/His, Arg/His genotypes and Arg/His genotypes were significantly different in the exposed group, but not in the healthy control group. MNBNC frequency is an important indicator to assess the level of CIN. The biological evidence has indicated that the *MAD1L1* Arg558His may indirectly lead to CIN by severely disturbing the function of the mitotic check point. As we know, the genetic effect of the variants on the important phenotype is modest, and it could be detected by a study with a sufficiently large sample size. However, when exposed to some pollutants like e-waste, the genetic effect of variants can emerge by the induction of a toxicant. Therefore, it can be concluded that *MAD1L1* Arg558His may contribute to CIN potentially through environmental exposure. It can also be speculated that the negative result in the healthy control group may be due to the relatively low statistical power of detecting the modest effect of the potential functional SNPs. Furthermore, the significant effect of *MAD1L1* Arg558His on the formation of aneuploidy was also observed in a previous study^29^. Future studies need to further dissect the genetic effect of *MAD1L1* Arg558His on chromosomal instability in healthy subjects with enough sample size. However, the negative effect of *MAD2L1* 84Leu/Met on the formation of aneuploidy as found in both the exposed and in the healthy control groups. Therefore, independent replication studies with a larger sample size are warranted to verify the negative result.

The present study has addressed some limitations, and it has some advantages. First, the sample size of the study was not very large, and the statistical power is relatively low for detecting the gene-to-gene interaction between *MAD1L1* Arg558His and *MAD2L1* Leu84Met, and the effect of *MAD1L1* Arg558His or *MAD2L1* Leu84Met on the formation of aneuploidy. Besides, because of the epidemiological background, we have not addressed the biological functional analyses to dissect the molecular mechanism underlying the significant statistical interaction between *MAD1L1* Arg558His, *MAD2L1* 84Leu/Met and smoking. Despite these limitations, our study emphasized that *MAD1L1* Arg558His and *MAD2L1* Leu84Met variations confer susceptibility to CRC in a Han Chinese population. Also, our findings highlight the point that two variations can significantly interact with smoking to enhance the risk of CRC. The information of the smoking level was detailed and complete, so the OR for *MAD1L1* Arg558His and *MAD2L1* Leu84Met that are stratified by different smoking levels were able to be estimated in the present study. In addition, the study showed that *MAD1L1* Arg558His may contribute to CIN potentially through the induction of environmental exposure. Nevertheless, follow-up studies are needed to uncover the biological mechanism behind the significant statistical interaction between *MAD1L1* Arg558His, *MAD2L1* 84Leu/Met and smoking, and further clarify the genetic effect of MAD1L1 Arg558His on chromosomal instability.

## Methods

### Subject

The present study included 710 CRC patients with newly diagnosed CRC and 735 cancer-free controls. Patients were consecutively recruited between January 1, 2010 and November 31, 2013 at Tongji Hospital of Huazhong University of Science and Technology (HUST), Wuhan, central China. Cancer-free individuals living in Wuhan city and its surrounding regions were randomly selected from a healthy screening conducted at the same hospital during the same time. All subjects were unrelated Han Chinese. The inclusion criteria for patients included histopathologically confirmed CRC, without previous chemotherapy or radiotherapy, and no restriction in regards to sex, age, or disease stage. The selection criteria for controls included cancer-free individuals and frequency matched to cases for sex and age within five years. At recruitment, written informed consent was obtained from each subject, and blood samples and demographic characteristics, including sex, age, and smoking habits, were collected by the interviewers. The detailed definitions of smoking status were described previously^30^. For smokers, the smoking level was determined by the number of packs per year smoked, which was calculated to indicate the cumulative cigarette dose level [packs per year = (cigarettes per day/20) × (years smoked)]. The median pack per year value of the controls was used as the cut-off point to categorize the light from the heavy smokers.

The participants who were included in the CBMN to estimate the genetic effect of two functional genetic variants on chromosomal instability were selected from a typical e-waste recycling site in southeast China as the exposure group and a nearby village (50 km away from the exposure site) without known sources of industrial pollution as the control group^31^. Seventy exposed subjects and 70 control subjects were randomly selected from these two groups. This study was conducted under the approval of the institutional review boards of Tongji Medical College of Huazhong University of Science and Technology.

### SNPs and genotyping

Genomic DNA was extracted from 2 ml of peripheral blood samples using the approved guideline of the Relax Gene Blood DNA System DP319-02 (Tiangen, Beijing China). Candidate SNPs were genotyped using PCR-based restriction fragment length polymorphism (RFLP) methods which were carried out in accordance with the approved guidelines, without knowledge of the case or control status of the subjects. The PCR primer pairs used for amplifying DNA containing the *MAD1L1* Arg558His or *MAD2L1* Leu84Met sites were described previously^16^. The PCR program was heating to 95 °C for 2 minutes followed by 35 cycles of 94 °C for 30 seconds, 63.5 °C (for *MAD1L1*) or 62 °C (for *MAD2L1*) for 30 seconds, 72 °C for 45 seconds, and a final elongation step of 72 °C for 10 minutes. Restriction enzymes *BstU*I or *AlwN*I (New England Biolabs, Beverly, Massachusetts, USA) were used to distinguish the *MAD1L1* Arg558His or *MAD2L1* Leu84Met genotypes, respectively. Quality control was monitored by including a 5% duplicate, with a 100% concurrence rate of the duplicate sets. The average call rate for the candidate SNPs genotyped was >99%.

Genomic DNA was extracted from the 2 ml of peripheral blood samples using the Relax Gene Blood DNA System DP319-02 (Tiangen, Beijing China). Candidate SNPs were genotyped using the TaqMan real-time polymerase chain reaction (PCR) Assay (Applied Biosystems, Foster city, CA) without knowledge of the case or control status of the subjects. The PCR program included heating to 95 °C for 10 minutes followed by 50 cycles of 92 °C for 15 seconds and 60 °C for 90 seconds. The ABI Prism 7900HT Sequence Detection System was applied to read the reacted plates and to analyze the endpoint fluorescence. Quality control was monitored by including a 5% duplicate and negative control, with a 100% concurrence rate of the duplicate sets. The average call rate for the candidate SNPs genotyped was >99%. All the experimental protocols were approved by TIANGEN BIOTECH (BEIJING) CO., LTD and New England Biolabs, MA, USA.

### MNBNCs frequency

70 independent exposed subjects and 70 control subjects were selected to determine the genotypes of *MAD1L1* Arg558His and *MAD2L1* Leu84Met, and the corresponding MNBNC frequency. The genotype method was described in the previous paragraph. The frequency of MNBNCs was detected by the method that was carried out in accordance with the approved guidelines and was described in detail in the previous study^32^. Briefly, 0.5 ml of a heparinized blood sample was added to 4.5 ml of medium with 15% fetal calf serum, 100 IU/mL penicillin and 20 μg/mL phytohemagglutinin (Sigma, St. Louis, MO, USA). After 44 hours of incubation and stimulation with phytohemagglutinin, cytochalasin B (Sigma, St. Louis, MO, USA) was added to the cultures at a final concentration of 6 μg/mL. Cells were harvested after 72 hours of incubation. Slides were prepared and stained with 10% Giemsa solution. The number of micronucleated binucleated cells in 1000 binucleated lymphocytes (MNBNCs frequency) was counted under a light-microscopy (Olympus C X21, Germany), according to the previously described criteria^33^.

### Statistical analysis

The Hardy-Weinberg equilibrium (HWE) for genotypes was assessed by a goodness-of-fit X2 test in the control groups. In the baseline analysis, the X2 test was applied to examine the differences between cases and controls in distribution of sex, age, smoking status and smoking level. The one-way ANOVA was used to determine the differences of MNBNC frequency for individuals with different genotypes. Co-dominant, dominant, and additive genetic models were assumed in the association analysis. Because minor allele frequencies (MAF) of MAD1L1 Arg558His and MAD2L1 84Leu/Met were 0.461 and 0.059 in controls, we calculated the power for the sample size of 710 patients and 735 controls as follows: for SNP with MAF of 0.059, the power for our sample size to detect an OR of 1.50 was 0.51; for SNP with MAF of 0.461, the power to detect an OR of 1.50 is 0.97. Interaction between SNPs was subsequently systematically investigated by a pair-wise analysis under multiplicative and additive interaction models. The P-values for multiplicative interaction were calculated using a multiplicative interaction term included in the multivariate logistic regression model in SPSS software. The P values for the additive interaction were calculated by a bootstrapping test of goodness-of-fit of the null hypothesis for no departure from an additive model vs. an alternative hypothesis for a departure from an additive model using Stata 10.0 (Stata Corporation, College Station, TX).

## Additional Information

**How to cite this article**: Zhong, R. *et al*. *MAD1L1* Arg558His and *MAD2L1* Leu84Met interaction with smoking increase the risk of colorectal cancer. *Sci. Rep*. **5**, 12202; doi: 10.1038/srep12202 (2015).

## Figures and Tables

**Table 1:** Distributions of select characteristics among cases and controls.

Variable	Controls (*n* = 735)	Cases (*n* = 710)	*P* value
	N (%)	N (%)	
Gender			0.520
Male	536(72.9)	507 (71.4)	
Female	199 (27.1)	203 (28.6)	
Age (y)			0.228
≤50	173 (23.5)	176 (24.8)	
51–60	233 (31.7)	249 (35.1)	
61–70	264(35.9)	219 (30.8)	
>70	65(8.8)	66 (9.3)	
Smoking status			<0.001
Nonsmoker	334 (45.4)	255 (35.9)	
Smoker	401 (54.6)	455 (64.1)	
Pack-years smoked			<0.001
≤24 pack-years	204(50.9)	164 (36.0)	
>24 pack-years	197 (49.1)	291 (64.0)	

**Table 2:** Genotype frequencies of *MAD1L1* and *MAD2L1* genotypes and their association with CRC risk.

Genotypes	Controls (%)	Cases (%)	OR (95% CI)†	*P* value
*MAD1*				
Arg/Arg	215 (29.3)	165 (23.2)	Reference	
Arg/His	363 (49.4)	346 (48.7)	1.24(0.96–1.60)	0.091
His/His	157 (21.4)	199 (28.0)	1.70 (1.26–2.28)	4.489 × 10^−4^
Dominant			1.38 (1.09–1.75)	0.008
Additive			1.30 (1.12–1.51)	4.634 × 10^−4^
*MAD2*				
Leu/Leu	650 (88.4)	591 (83.2)	Reference	
Leu/Met	84 (11.4)	114 (16.1)	1.44 (1.06–1.96)	0.020
Met/Met	1 (0.1)	5 (0.7)	4.74 (0.55–40.79)	0.156
Dominant			1.48 (1.09–2.01)	0.011
Additive			1.49 (1.11–2.00)	0.007

^†^ORs and 95% CIs were calculated by unconditional logistic regression after adjusting for sex, age and smoking status.

**Table 3:** Stratification and interaction analysis between *MAD1L1* and *MAD2L1* genotypes associated with CRC risk.

Genotypes		Controls(*n* = 735)	Cases(*n* = 710)	
*MAD1*	*MAD2*	N (%)	N (%)	OR (95% CI)†; *P*
Arg/Arg	Leu/Leu	196 (26.7)	139 (19.6)	Reference
Arg/Arg	Leu/Met+Met/Met	19 (2.6)	26 (3.7)	1.90 (1.00–3.59); 0.049
Arg/His	Leu/Leu	317 (43.1)	294 (41.4)	1.32 (1.01–1.73); 0.045
Arg/His	Leu/Met+Met/Met	46 (6.3)	52 (7.3)	1.51 (0.95–2.38); 0.081
His/His	Leu/Leu	137 (18.6)	158 (22.3)	1.67 (1.22–2.30); 0.002
His/His	Leu/Met+Met/Met	20 (2.7)	41 (5.8)	2.92(1.63–5.24); 3.140 × 10^−4^
	*P*_mult /_ *P*_add_		0.361/0.708	

^†^ORs and 95% CIs were calculated by unconditional logistic regression after adjusting for sex, age and smoking status.

**Table 4:** Stratified and interaction analysis between *MAD1L1*, *MAD2L1* genotypes and smoking status associated with the risk of CRC.

Smoking status	*MAD1*Arg/Arg		*MAD1*Arg/His+ His/His		*P*_mult /_ *P*_add_
	Cases/controls	OR (95% CI)†; *P*	Cases/controls	OR (95% CI)†; *P*	
nonsmoker	52/95	Reference	203/239	1.52 (1.03–2.25); 0.036	0.019/0.557
smoker	113/120	2.22 (1.40–3.52); 0.001	342/281	2.88 (1.91–4.35); 4.785 × 10^−7^	
≤24 pack-years	49/65	1.76 (1.03–2.99); 0.038	115/139	1.91(1.21–3.01); 0.005	
>24 pack-years	64/55	2.87 (1.69–4.87); 9.907 × 10^−5^	227/142	3.96(2.56–6.13); 7.299 × 10^−10^	
	*MAD2* Leu/Leu		*MAD2* Leu/Met+ Met/Met		
Smoking status	Cases/controls	OR (95% CI)†; *P*	Cases/controls	OR (95% CI)†; *P*	*P*_mult /_ *P*_add_
nonsmoker	209/299	Reference	46/35	1.74 (1.08–2.82); 0.023	0.016/0.736
smoker	382/351	1.99(1.50–2.63); 1.432 × 10^−6^	73/50	2.63 (1.71–4.05); 1.132 × 10^−5^	
≤24 pack-years	131/176	1.35 (0.97–1.87); 0.075	33/28	2.03 (1.17–3.54); 0.012	
>24 pack-years	251/175	2.74 (2.01–3.74); 2.460 × 10^−10^	40/22	3.48 (1.96–6.21); 2.282 × 10^−5^	

^†^ORs and 95% CIs were calculated by unconditional logistic regression after adjusting for sex and age.

*P*_mult_ was calculated using the multiplicative interaction term. *P*_add_ was calculated using the additive interaction model.
